# MiR-21, MiR-29a, GATA4, and MEF2c Expression Changes in Endothelin-1 and Angiotensin II Cardiac Hypertrophy Stimulated Isl-1^+^Sca-1^+^c-kit^+^ Porcine Cardiac Progenitor Cells In Vitro

**DOI:** 10.3390/cells8111416

**Published:** 2019-11-09

**Authors:** Katrin Zlabinger, Andreas Spannbauer, Denise Traxler, Alfred Gugerell, Dominika Lukovic, Johannes Winkler, Julia Mester-Tonczar, Bruno Podesser, Mariann Gyöngyösi

**Affiliations:** 1 Medical University of Vienna, Department of Cardiology, 1090 Vienna, Austria; andreas.spannbauer@meduniwien.ac.at (A.S.); denise.traxler-weidenauer@meduniwien.ac.at (D.T.); Alfred.gugerell@meduniwien.ac.at (A.G.); dominika.lukovic@meduniwien.ac.at (D.L.); johannes.winkler@meduniwien.ac.at (J.W.); Julia.mester-tonczar@meduniwien.ac.at (J.M.-T.); 2 Medical University of Vienna, Department of Biomedical Research, 1090 Vienna, Austria; Bruno.podesser@meduniwien.ac.at

**Keywords:** porcine cardiac progenitor cells, cardiac hypertrophy, cardiac remodeling, Isl-1, angiotensin II, endothelin 1, MEF2c, GATA4, miR-21, miR29a

## Abstract

Cost- and time-intensive porcine translational disease models offer great opportunities to test drugs and therapies for pathological cardiac hypertrophy and can be supported by porcine cell culture models that provide further insights into basic disease mechanisms. Cardiac progenitor cells (CPCs) residing in the adult heart have been shown to differentiate in vitro into cardiomyocytes and could contribute to cardiac regeneration. Therefore, it is important to evaluate their changes on the cellular level caused by disease. We successfully isolated Isl1^+^Sca1^+^cKit^+^ porcine CPCs (pCPCs) from pig hearts and stimulated them with endothelin-1 (ET-1) and angiotensin II (Ang II) in vitro. We also performed a cardiac reprogramming transfection and tested the same conditions. Our results show that undifferentiated Isl1^+^Sca1^+^cKit^+^ pCPCs were significantly upregulated in GATA4, MEF2c, and miR-29a gene expressions and in BNP and MCP-1 protein expressions with Ang II stimulation, but they showed no significant changes in miR-29a and MCP-1 when stimulated with ET-1. Differentiated Isl1^+^Sca1^+^cKit^+^ pCPCs exhibited significantly higher levels of MEF2c, GATA4, miR-29a, and miR-21 as well as Cx43 and BNP with Ang II stimulation. *pMx-MGT*-transfected Isl1^+^Sca1^+^cKit^+^ pCPCs showed significant elevations in MEF2c, GATA4, and BNP expressions when stimulated with ET-1. Our model demonstrates that in vitro stimulation leads to successful Isl1^+^Sca1^+^cKit^+^ pCPC hypertrophy with upregulation of cardiac remodeling associated genes and profibrotic miRNAs and offers great possibilities for further investigations of disease mechanisms and treatment.

## 1. Introduction

Cardiac hypertrophy is characterized by the increase of the heart size, as well as cardiomyocyte size, and mass in response to pathological and physiological mechanisms [1,2,3]. Pathological cardiac remodeling is a non-reversible process that describes changes on the cellular level as well as changes in shape, size, and heart function that are caused by pathologic stimuli [4]. The interaction of apoptotic cells, hypertrophic cardiomyocytes, and proliferation of fibroblasts leads to fibrous tissue formation with increased myocardial stiffness [2,5]. In consequence, volume overload develops, which leads to further cell damage and cardiomyocyte loss [6]. On the cellular level, heart tissue comprises cardiomyocytes, fibroblasts, and endothelial cells, which are based in the extracellular matrix [2,6]. Furthermore, specialized adult cardiac progenitor cell (CPC) populations can be found in mammals in interstitial places of the heart near cardiomyocytes and are known to express progenitor cell markers like stem cell antigen-1 (Sca-1), insulin gene enhancer protein-1 (Isl-1), or the stem cell growth factor receptor c-kit [7,8]. In an undifferentiated state they are able to proliferate and differentiate into only restricted related families of cells from adult tissue to replace damaged cells [8]. Isl-1-positive CPCs originate from the second heart field in cardiac development, and they have been shown to give rise to cardiac cells of different lineages such as cardiomyocytes, smooth muscle cells, and vascular endothelial cells [9,10]. They are part of the outflow tract, right/left atrium, and ventricle as well as vessels from the inflow tract, but they are reported to be very rare in proportion to other cells [10,11,12]. Isl-1 progenitors have been shown to play a substantial role in cardiac development. As documented by Cai et al., Isl-1 deficiency in mice caused malformations and functional loss [12]. Together with zinc finger transcription factor GATA4 and homeobox protein transcription factor NKX2.5, Isl-1 targets the myocyte-specific enhancer factor 2C (MEF2c) transcription factor directly and is, therefore, required for the differentiation of pCPC into cardiomyocytes or smooth muscle cells [10,12,13,14]. In the development of cardiac hypertrophy, GATA4 plays a role by targeting genes of pressure overload associated proteins, such as angiotensin II type 1a receptor [15], myosin heavy and light chains [16,17,18], troponins [19,20,21], brain natriuretic peptide (BNP), atrial natriuretic peptide (ANP) [22,23] and many more. In response to hypertrophic stimuli, GATA4 expression is increased and vice versa [24,25,26]. Since MEF2c is connected to GATA4 and Isl-1, other groups have documented the upregulation in gene expression in connection with cardiac hypertrophy [27,28]. Furthermore, the contribution of microRNAs (miRNAs) in cardiac diseases has been intensively investigated in the last few years [29]. MiRNAs are small RNAs that do not code for proteins but play a significant role in gene expression regulation and RNA silencing by base-pairing with complementary mRNA sequences and are, therefore, part of several physiological and pathological mechanisms [6,29]. There is significant evidence that miR-21, miR-23a, or miR208a overexpression leads to pathological cardiac hypertrophy [30,31,32], and decreased levels of miR-24, miR-29, miR-199, and miR-320 are associated with myocardial infarction and hypoxia-induced responses [29,33,34,35,36]. Recent studies reported that miR-29a upregulation is involved in the development of cardiac hypertrophy and is directly associated with fibrosis [37,38].

For the investigation of pathological processes and detection of potential new therapeutic agents, cardiac hypertrophy can be simulated in in vitro models using different stimulating agents that mimic hypertrophic pathways through targeting transcription factors. Cardiac hypertrophy cell culture models can be elicited by stimulating cells with agents such as endothelin-1 (ET-1) or angiotensin II (Ang II) [39,40,41,42,43]. ET-1 is used as a vasoconstricting agent that also stimulates the renin–angiotensin–aldosterone system. It positively affects the contraction (inotropy) and beating rate (chronotropy) of the heart and acts as an activator of GATA4 in cardiac cells [44,45,46,47]. Stimulation with ET-1 leads to an increase in myocyte width and overall muscle mass, both indicators of cardiac hypertrophy [42]. Angiotensin II is part of the renin–angiotensin system and has also been used to stimulate cardiac hypertrophy in cardiomyocytes in vitro by mediating hypertrophic growth via binding of GATA4 [15,43].

Cardiomyocytes from different origins and species, such as mouse or rat primary cardiomyocytes [25,27,48,49] or human iPS derived cardiomyocytes [50,51], can be used for the establishment of in vitro cardiac hypertrophy models. Nevertheless, the porcine heart is currently one of the best available translational animal models for cardiovascular research despite it being cost- and time-intensive [52]. Therefore, there is an urgent need for porcine cardiac in vitro models to investigate potential new drugs or therapies before going in vivo. Interestingly, in spite of growing evidence of the necessity of a porcine translational model, there is a consequent lack of porcine cardiac cells for in vitro experiments. Hence, we established the isolation and culture of CPCs from porcine heart tissue to stimulate them in vitro with ET-1 and Ang II. We also investigated the effect of transfection with retroviral expression vector with cardiac reprogramming genes (*pMx-MGT*, inserting MEF2c, Tbx5, GATA4 genes) on the hypertrophic outcome. We evaluated pCPC hypertrophy by measuring the mean cell area, quantification of cellular proteins with immunofluorescence staining, and mRNA and miRNA expression focusing on GATA4 and MEF2c as well as miR-21 and miR-29a.

## 2. Materials and Methods

### 2.1. Isolation of Porcine Cardiac Progenitor Cells from Heart Tissue

Porcine hearts were explanted from euthanized pigs (*n* = 10), stored in 1× Dulbecco’s phosphate-buffered saline (1× D-PBS, Sigma Aldrich, St. Louis, MO, USA) supplemented with 2% penicillin–streptomycin (PenStrep, Sigma Aldrich, St. Louis, MO, USA), and immediately delivered to the laboratory. Under sterile conditions, heart muscle tissue from the left atrium, left ventricle, and apex was excised and used for the isolation of porcine cardiac progenitor cells (pCPC) from the heart muscle stem cell niche. The pieces of heart muscle were minced into chunks approximately 2 mm^2^ in size and were transferred to gentleMACS C-tubes (Miltenyi Biotec, Bergisch Gladbach, Germany), which were filled with 5 mL of 0.02% collagenase II (Worthington Biochemical Corp., Lakewood, New Jersey, USA) in M199 medium (Sigma Aldrich, St. Louis, MO, USA). The filled C-tubes were placed into the gentleMACS dissociator (Miltenyi Biotec, Bergisch Gladbach, Germany), and cells were dissociated with the appropriate dissociation protocol (m_neonatal_heart). Afterwards, the tubes were incubated for 20 min at 37 °C in a water bath to start the collagenase digestion. The cell mixture was filtered through a 100 µm filter (Falcon cell strainer, Corning Life Sciences, Corning, NY, USA), washed with Earle’s balanced salt solution (EBSS, Gibco/Thermo Fisher Scientific, Waltham, MA, USA), and centrifuged for 5 min at 280 rcf. Cell supernatant was discarded, and the cells were resuspended in 30 µL EBSS, filtered through a 70 µm filter (Falcon cell strainer, Corning Life Sciences, Corning, NY, USA), and centrifuged for another 5 min at 1500 rpm. The cell pellet was then resuspended in pCPC medium consisting of M199 and Dulbecco’s modified Eagle’s medium (DMEM, Sigma Aldrich, St. Louis, MO, USA) in a 1:1 ratio supplemented with 10% fetal bovine serum (FBS, Biochrom Ltd., Cambridge, Great Britain) and 1% pen–strep and cultivated for 24 h in cell culture flasks. The day after cell seeding, culture medium was refreshed to get rid of cell debris. Usually, one week after isolation, cells start to form single colonies. pCPCs are plastic adherent cells that were cultivated in pCPC medium (see above). Media was changed every 2–3 d, and the cells were separated once a week, dependent on the splitting rate of 1:3–1:5.

### 2.2. Characterization of pCPCs

#### 2.2.1. Immunofluorescence Staining

Porcine cardiac progenitor cells form different populations that express different markers. They are known to express progenitor cell markers such as stem cell antigen-1 (Sca-1), islet-1 (Isl-1), or the stem cell growth factor receptor c-kit, besides other cardiac progenitor cell related markers. After cell isolation and propagation, cells were stained for progenitor cell and cardiomyocyte related markers, Isl-1 (1:100; biorbyt, Cambridge, GB), Sca-1 (1:100; Thermo Fisher Scientific, Waltham, MA, USA), cTNT (1:400; abcam, Cambridge, GB), Cx43 (1:1000; abcam, Cambridge, GB), and αSMA (1:200; abcam, Cambridge, GB), using indirect immunofluorescence staining. pCPCs were seeded to 96-well plates at a concentration of 1 × 10^4^ cells per well and cultivated overnight in a CO_2_ incubator at 37 °C. Once the cells reached about 90% confluence, they were ready for the immunofluorescence staining procedure. Medium was removed, wells were washed twice with 1× D-PBS, and they were fixed for 15 min with a 2.5% paraformaldehyde (powder dissolved in aqua dest., Carl Roth GmbH, Karlsruhe, Germany) solution in 1× D-PBS. The primary antibodies were diluted in 3% nonfat dry milk (Sigma Aldrich, St. Louis, MO, USA) with 0.1% Triton X-100 (Sigma Aldrich, St. Louis, MO, USA) according to the manufacturers’ protocol, and they were added to the cells for overnight incubation at 4 °C. The next day, the antibody solution was removed from each well, washed twice with 1× D-PBS, and the secondary antibody was added and incubated for an hour in the dark at room temperature. Cells were counterstained with Hoechst (1:5000, Sigma Aldrich, St. Louis, MO, USA) and phalloidin (1:40, Thermo Fisher Scientific, Waltham, MA, USA), and they were observed with an Olympus IX83 Inverted Microscope using cellSens imaging software (Olympus, Tokio, Japan)

#### 2.2.2. Real-Time qPCR

For RNA isolation of the cells, medium was removed, and cells were washed with 1× D-PBS. Afterwards, 350 µL RLT (RNeasy Lysis) buffer (Qiagen, Hilden, Germany) supplemented with β-mercaptoethanol (1:1000, Sigma Aldrich, St. Louis, MO, USA) was added to the well, pipetted carefully up and down, transferred to a 1.5 mL reaction tube, and stored at −20 °C until all samples were collected for the RNA isolation procedure. Cell suspension (350 µL) was transferred to a QIAshredder tube (Qiagen, Hilden, Germany) and centrifuged for 2 min at high speed in an Eppendorf microcentrifuge. For RNA isolation, the eluate was processed with a QIACube automated RNA isolation machine with the miRNeasy Mini Kit (Qiagen, Hilden, Germany). The program “purification of total RNA, including small RNAs, from tissues and cells” was set on the QIACube, and it was assembled with protocol-dependent reagents, tubes, and tips. RNA was eluted in RNase-free water and was checked for RNA concentration and quality on a NanoDrop 2000 microvolume spectrophotometer (Thermo Fisher Scientific, Waltham, MA, USA). The dilution of each sample was calculated to obtain a final concentration of 500 ng/µL cDNA after reverse transcriptase (RT) procedure. We used the RT Kit from Qiagen. Samples were diluted with varying amounts of RNase-free water together with 4 µL gDNA wipeout buffer to a total volume of 28 µL in 2 mL reaction tubes. The mixture was incubated for 2 min at 42 °C, then placed on ice. In the meantime, a mastermix (MM) was prepared (2 µL reverse transcriptase + 8 µL RT Buffer + 2 µL RT primer), added to the template RNA samples, and incubated for 15 min at 42 °C. Afterwards, reverse transcriptase was inactivated with a 3 min incubation at 95 °C. The cDNA was either used directly for qPCR analyses or stored at −20 °C. Primers (Table 1) were dissolved in RNAse-free water to a concentration of 100 µM. For the qPCR procedure (SYBR Green qPCR Kit, Qiagen), primers had to be further diluted to a concentration of 10 µM. Samples were diluted down to a concentration of 5 ng/µL (from 40 µL stock to 200 µL). Combining aliquots from all samples generated a standard sample. The standard curve samples were diluted in a 1:3 ratio. The master mix was prepared for each target (10 µL mastermix, 2 µL primer forward, 2 µL primer reverse) and added to each dedicated well in a 96-well PCR plate. Six microliters of sample cDNA (or gDNA), standard, and blank was added in duplicate to each MM-prepared well. The plate was sealed with a transparent PCR foil and was centrifuged for 15 s before it was placed into the Quantsudio 5 (Thermo Fisher Scientific, Waltham, MA, USA) real-time qPCR-cycler to start the run.

#### 2.2.3. Transfection with *pMx-MGT*

pCPCs were transfected with the *pMx-MGT* plasmid (retroviral expression vector for direct cardiac reprogramming: MEF2c, GATA4, Tbx5) using a Lipofectamine LTX Transfection Kit. *pMx-MGT* was a gift from Li Qian (Addgene plasmid #111810; http://n2t.net/addgene:111810; RRID: Addgene_111810) [53] and was extracted from a bacterial stock using a plasmid DNA purification kit from Qiagen (QIAGEN Plasmid Midi Kit). According to the manufacturers’ protocol, 1 µg of plasmid diluted with Lipofectamine LTX in OptiMEM medium (Thermo Fisher Scientific, Waltham, MA, USA) was used for the transfection in 6-well plates and accordingly downscaled or upscaled depending on the well size. Cell medium was refreshed after 72 h of transfection.

#### 2.2.4. Induction of Hypertrophy in pCPC

pCPCs were seeded into 48-well plates, and for each condition, 3 wells were used and pooled afterwards to get a sufficient amount of mRNA/miRNA. For the staining of cells, they were seeded to 96-well plates. Williams’ E medium was supplemented with 10% FBS and 1% penicillin/streptomycin solution (SWE medium) and used throughout the cultivation time. Once the cells reached 70–90% confluence, chemical-induced hypertrophy was implemented. Angiotensin II was dissolved to a stock solution of 100 nM/mL in SWE medium. From this stock, Ang II was diluted to a concentration of 100 nM in SWE medium and added to the cells for 24 h. ET-1 was dissolved to a concentration of 1 µL/mL in aqua dest. and added in a concentration of 2.5 nM to the cells for 24 h.

### 2.3. Measurement of Hypertrophy-Induced Protein Expression and Cell Size

pCPCs were seeded to 96-well plates, hypertrophy was induced, and cells were fixed with 2.5% paraformaldehyde. Immunofluorescence staining for monocyte chemotactic protein 1 (MCP-1), connexin 43 (Cx43), and brain natriuretic peptide (BNP) was performed as described in 2.2.1, and mean integrated intensity (pixel/cell) was measured with Cell Profiler 3.1.9 for Windows and compared to untreated cells. Cell size was also measured using Cell profiler, evaluating the mean cell area depicted by phalloidin actin filament staining.

### 2.4. Quantification of mRNA and miRNA Gene Expression by Real-Time qPCR

Total RNA isolation, RT, and real-time qPCR was performed as described in Section 2.2.2. For the miRNA qPCR, a Universal Primer was used instead of a second primer to build the complementary sequence for the inserted miRNA sequences (Table 2).

### 2.5. Statistics

All graphs were arranged and statistics were carried out using Graph Pad Prism Software (Version 5.01, GraphPad, San Diego, CA, USA). Normal distribution of data was tested with D’Agostino–Pearson tests. Differences between hypertrophic and control pCPCs were tested by sign-paired t-tests for normally distributed samples and Wilcoxon signed-rank tests for samples that were not normally distributed.

## 3. Results

### 3.1. Isolation and Characterizaton of Isl1^+^Sca1^+^ckit^+^ pCPC

**Figure 1:**
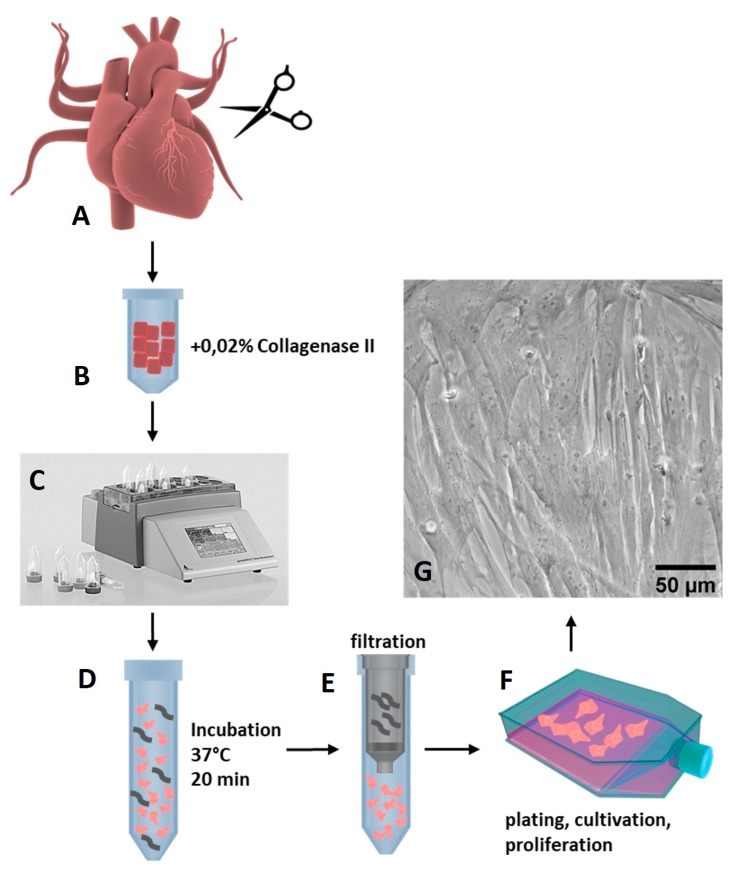
Isolation of pCPCs with collagenase digestion. (**A**) Tissue from the left atrium, left ventricle, and apex from pig hearts was excised into small chunks and transferred to (**B**) gentleMACs C Tubes together with 0.02% collagenase II solution in medium. (**C**) The tissue was mechanically dissociated on a gentleMACS dissociator and (**D**) incubated for 20 min at 37 °C in a water bath with gentle agitation. (**E**) The cell solution was filtered, (**F**) and isolated cells were plated to T75 flasks. (**G**) After one week, cells reached confluency and were further expanded and cryopreserved.

pCPCs were isolated from explanted healthy pig hearts (*n* = 10) that we received from the Department of Biomedical Research of the Medical University of Vienna. The ethical guidelines for animal care were followed. The animals were between 4–6 months old and weighed 30–50 kg. As depicted in Figure 1, the isolation of pCPC was obtained using a 0.02% collagenase II (Worthington Biochemical Corp., Lakewood, New Jersey, USA) digestion method in combination with mechanical disruption on a gentleMACS dissociatior (Miltenyi Biotec, Bergisch Gladbach, Germany).

**Figure 2:**
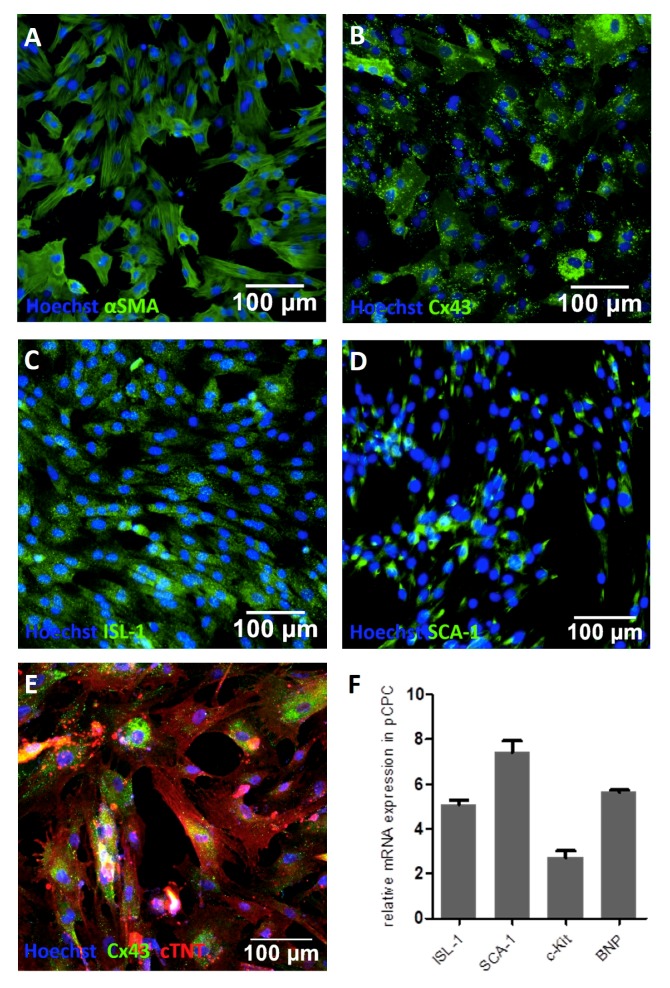
Characterization of pCPCs. Immunofluorescence staining revealed the presence of proteins associated with the cardiac cell lineage such as (**A**) αSMA, (**B**) Cx43, and (**E**) cTNT (depicted in red). Cardiac progenitor cell associated markers like (**C**) Isl-1 and (**D**) Sca-1 stained positive. (**F**) Relative gene expression further proved the presence of genes encoding for Isl-1, Sca-1, c-kit, and BNP.

pCPCs grew in fibroblast-like colonies (Figure 1G), proliferated relatively rapidly, and could be first subcultivated after one week of cultivation. pCPCs form different populations that express different markers. They are known to express progenitor cell markers such as stem cell antigen-1 (Sca-1), islet-1 (Isl-1), or the stem cell growth factor receptor c-kit, besides other cardiac progenitor cell related markers. As seen in Figure 2, immunofluorescence staining of the isolated pCPC population revealed positive expressions for Isl-1 (Figure 2C, green) and Sca-1 (Figure 2D, green). Cardiac lineage associated proteins such as Cx43 (Figure 2B, green) and cTNT (Figure 2E, red) were also detected. αSMA, an actin protein that is associated with myofibroblastsis and also highly expressed in stem cells, was also ascertained (Figure 2A, green). The relative gene expression, normalized to the housekeeping gene *ACTB*, was examined. Depicted in Figure 2F, genes encoding for ISL-1 (relative gene expression = 5 ± 0.36), SCA-1 (7.4 ± 0.88), c-Kit (2.72 ± 0.77), and BNP (5.63 ± 0.22) could be detected in different manifestations, proving the successful isolation of Isl1^+^Sca1^+^ckit^+^ pCPC.

### 3.2. Induction of Chemical Hypertrophy with Ang II and ET-1 Results in Hypertrophic Growth of Isl1^+^Sca1^+^ckit^+^ pCPC

**Figure 3:**
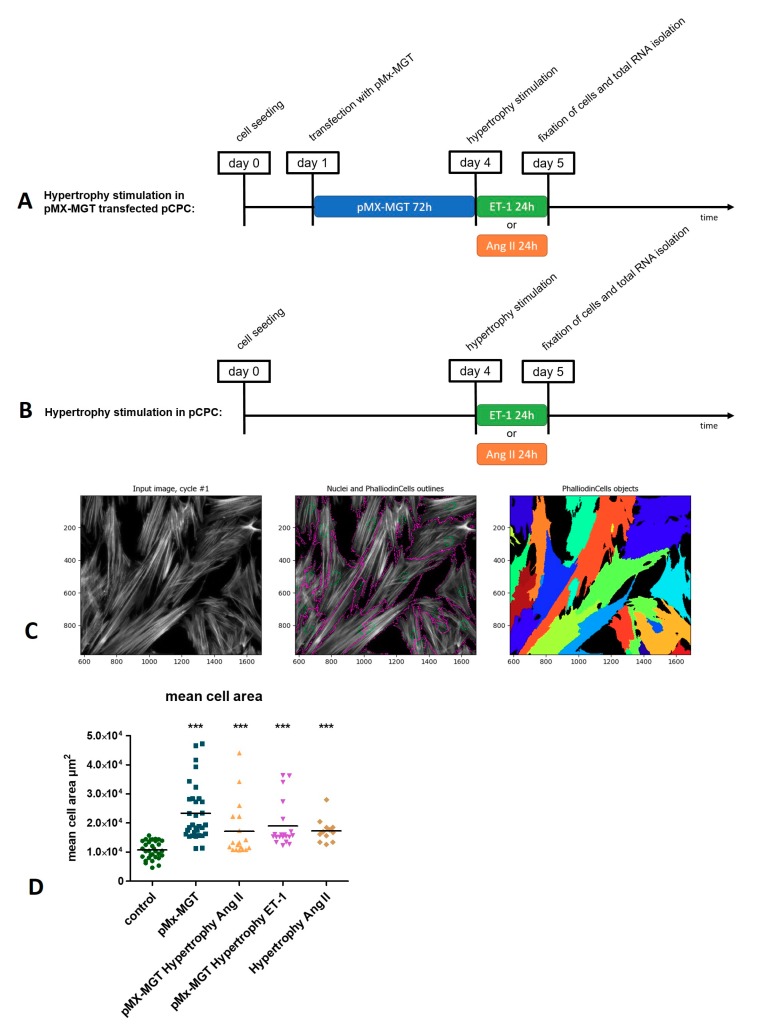
(**A**) Time course of hypertrophy induction of *pMx-MGT*-transfected Isl1^+^Sca1^+^ckit^+^ pCPCs. Cells were seeded at day 0; the next day they were transfected; on day 4, hypertrophy was induced, either with ET-1 or Ang II; and on day 5, the cells were fixed, and total RNA was isolated. (**B**) Depicts the time course of the hypertrophy induction without prior transfection. (**C**) Method of mean cell area (µm^2^) measurements using cell profiler software. Cell nuclei are marked with green, cytoplasm with purple outlines, and the mean cell area of each cell is represented in multiple colors. Mean cell area differences are depicted in (**D**).

As described in the experimental setup in Figure 3A,B, Isl1^+^Sca1^+^ckit^+^ pCPCs were either transfected with a cardiac reprogramming plasmid *pMx-MGT* (for 72 h) and then stimulated with Ang II or ET-1 (Figure 3A), or just stimulated with Ang II or ET-1 for 24 h. For the evaluation of cell size, the stimulated cells were stained with phalloidin, which binds to f-actin filaments and makes it possible to visualize the whole cell cytoplasm. As illustrated in Figure 3C, we used cell profiler imaging software for measuring the mean cell area with a special algorithm that combines primary objects (Hoechst-stained nuclei, green circles) with secondary objects (phalloidin-stained cytoplasm, purple outlines) enabling the identification of each single cell and measurement of the mean cell area occupied in the picture. The mean cell areas from the different treated groups were compared to the control cells (Figure 3D), which exhibited a mean cell area of 1.07 × 10^4^ ± 0.3 µm^2^. There was a significant increase in all groups, including *pMx-MGT*-transfected cells with a mean cell area of 2.34 × 10^4^ ± 0.97 µm^2^ (*p* < 0.0001); *pMx-MGT*-transfected cells with Ang II hypertrophy with a mean cell area of 1.71 × 10^4^ ± 0.93 µm^2^ (*p* < 0.0001); *pMX-MGT*-transfected cells with ET-1 hypertrophy with a mean cell area of 1.93 × 10^4^ ± 0.79 µm^2^ (*p* < 0.0001); and cells with Ang II hypertrophy with a mean cell area of 1.73 × 10^4^ ± 0.41 µm^2^ (*p* = 0.0005).

### 3.3. Cardiac Reprogramming of pCPCs with pMx-MGT or Ang II Induced Hypertrophy Alone Leads to Increase of Intracellular MCP-1

**Figure 4:**
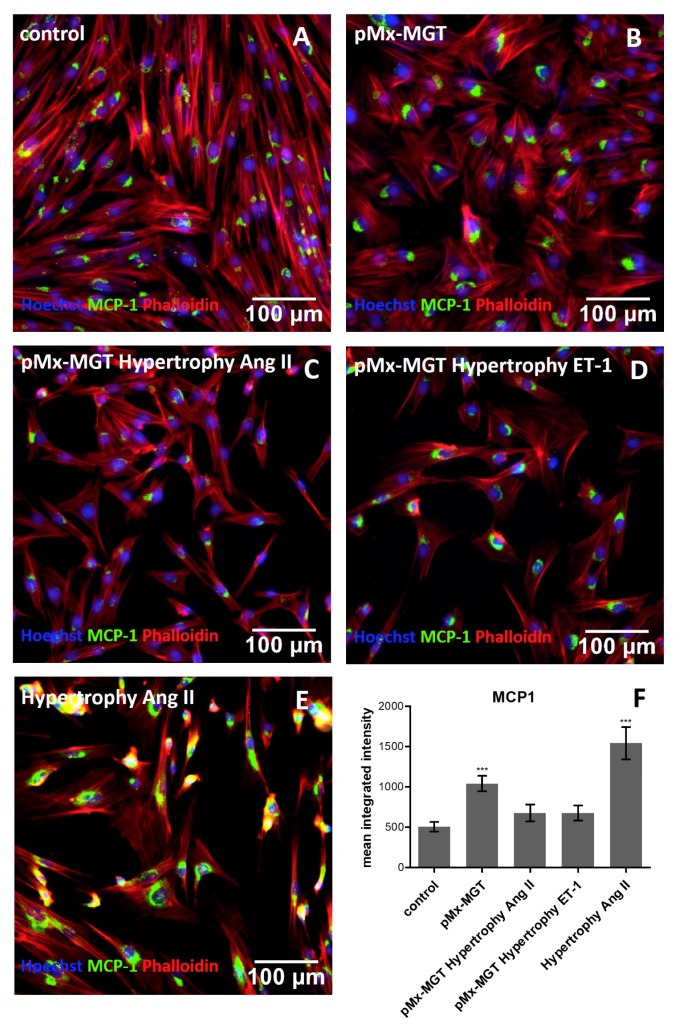
Immunofluorescence staining of MCP-1. (**A**) Control cells exhibit a mean integrated intensity of MCP-1 (green) of 505.1 ± 260.8 pixels/cell (**F**). Nuclei are counterstained with Hoechst (blue) and cytoplasm with phalloidin (red). (**B**) *pMx-MGT*-transfected Isl1^+^Sca1^+^ckit^+^ pCPCs had a significant increase in MCP-1 expression (1040.0 ± 430.9 pixels/cell; f) (**C**,**D**); *pMx-MGT* Ang II and *pMX-MGT* ET-1 cells showed no significant change in MCP-1 expression (676.3 ± 421 pixels/cell; 675.5 ± 409.1 pixels/cell; (**F**)). (**E**) Ang II stimulated Isl1^+^Sca1^+^ckit^+^ pCPCs expressed a significantly higher MCP-1 integrated intensity with a *p* = 0.0001 (1541.0 ± 880.9 pixels/cell). *** *p* ≤ 0.001.

We wanted to evaluate whether the MCP-1 level increased in response to hypertrophic stimulation in Isl1^+^Sca1^+^ckit^+^ pCPCs. Using a cell profiler pipeline for the measurement of object intensity, the MCP-1 alexa fluor 488 mean integrated intensity in each cell (pixel/cell) was determined and compared to the control with 505.1 ± 260.8 pixels/cell (Figure 4A,F). The *pMx-MGT*-transfected cells without hypertrophic stimulus exhibited a significantly higher expression of the mean integrated intensity of MCP-1 with 1040.0 ± 430.9 pixels/cell (*p* = 0.001) shown in Figure 4B,F. There was no significant change in the expression of *pMx-MGT* Ang II cells (676.3 ± 421.3 pixels/cell; Figure 4C,F) and *pMx-MGT* ET-1 cells (675.5 ± 409.1 pixels/cell; Figure 4D,F), but a highly significant upregulation of MCP-1 was measured in the Isl1^+^Sca1^+^ckit^+^ pCPCs stimulated with Ang II (1541.0 ± 880.9 pixels/cell, *p* = 0.0001; Figure 4E,F).

### 3.4. Cx43 is Upregulated in pMx-MGT-Transfected and Ang II Stimulated Isl1^+^Sca1^+^ckit^+^ pCPCs

**Figure 5:**
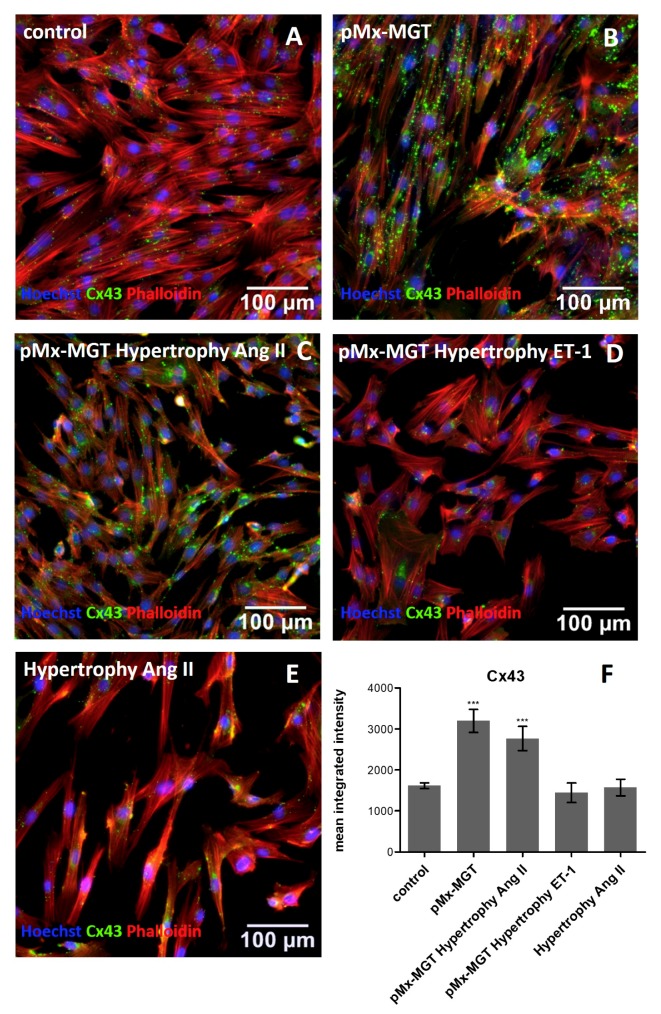
Immunofluorescence staining of Cx43. (**A**) Control cells with mean integrated intensity of 1616.0 ± 315.0 pixels/cell Cx43 (green, (**F**)); (**B**,**C**) significant changes in *pMx-MGT*-transfected Isl1^+^Sca1^+^ckit^+^ pCPCs (3196.0 ± 1224.0 pixels/cell, *p* = 0.0001) and in combination with Ang II stimulation (2766.0 ± 1324.0 pixels/cell, *p* = 0.0004). Mean integrated intensities are displayed in (**F**). (**D**,**E**) Cx43 intensities in *pMx-MGT* hypertrophy ET-1 (1443.0 ± 977.8 pixels/cell) and hypertrophy Ang II (1572.0 ± 905.5 pixels/cell) cells were not significantly changed compared to control cells. *** *p* ≤ 0.0004.

Cx43 is involved in cell communication and regulation of cell death and differentiation. The mean integrated intensity measured in the control cells was 1616 ± 315 pixels/cell (Figure 5A,F) and significantly changed when cells were transfected with cardiac reprogramming plasmid *pMx-MGT* (3196 ± 1224 pixels/cell, *p* = 0.0001, Figure 5B,F) or in combination with angiotensin II stimulation (2766 ± 1324 pixels/cell, *p* = 0.0004, Figure 5C,F). There was no change in expression when Isl1^+^Sca1^+^ckit^+^ pCPCs were transfected and stimulated with ET-1 (1443 ± 977.8 pixels/cell; Figure 5D,F) or stimulated with Ang II (1572 ± 905.5 pixels/cell; Figure 5E,F).

### 3.5. Increased Cellular BNP Expression Confirms Cardiac Hypertrophy

**Figure 6:**
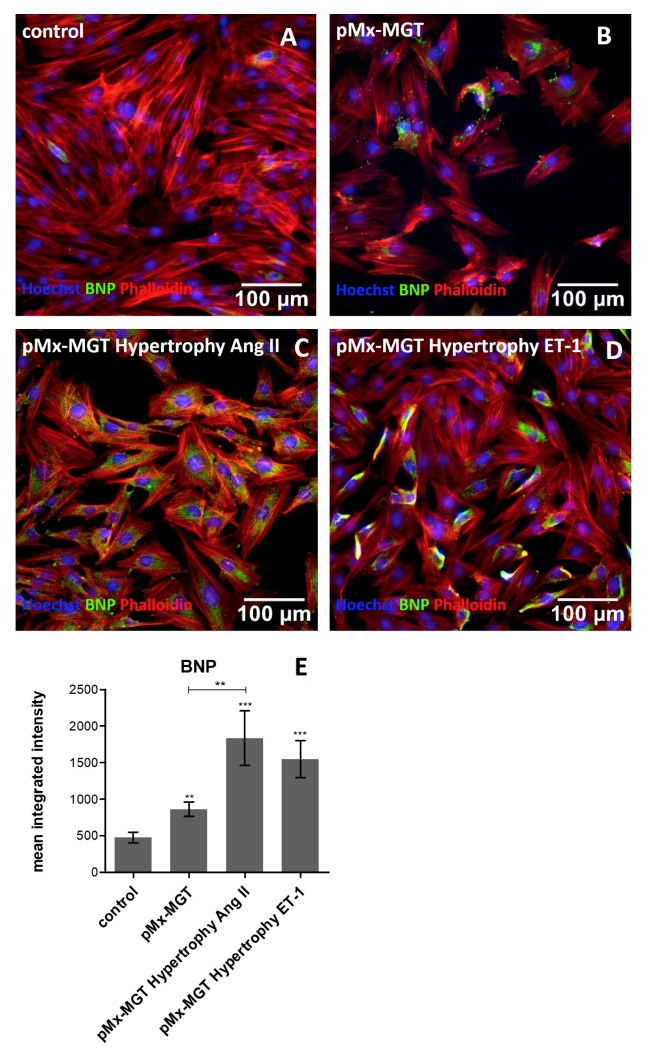
Immunofluorescence staining of BNP (green), cell nuclei (blue), and phalloidin (red). (**A**) Depicts BNP expression in control cells with a mean integrated intensity of 476.3 ± 328.6 pixels/cell. An increased BNP expression could be detected in (**B**) *pMx-MGT*-transfected pCPCs (862.2 ± 450.5 pixels/cell; *p* < 0.05), (**C**) *pMx-MGT* hypertrophy Ang II (1835.0 ± 1451.0 pixels/cell, *p* = 0.0006), and (**D**) *pMx-MGT* hypertrophy ET-1 (1547.0 ± 1128.0 pixels/cell, *p* = 0.0002). (**E**) Shows mean integrated intensities in all groups compared to control cells.

BNP, a frequently used clinical biomarker marker, is upregulated in cardiac remodeling processes and promotes cardiac fibrosis [54,55]. Our results confirm the assumption that chemical hypertrophy induction leads to higher expression of intracellular BNP. As seen in Figure 6A,F, the expression was much lower in the Isl1^+^Sca1^+^ckit^+^ pCPC control cells (476.3 ± 328.6 pixels/cell) compared to *pMx-MGT* hypertrophy Ang II (1835.0 ± 1451.0 pixels/cell, *p* = 0.0006; Figure 6C,E) and *pMx-MGT* hypertrophy ET-1 (1547.0 ± 1128.0 pixels/cell, *p* = 0.0002; Figure 6D,E) stimulated cells. The *pMx-MGT*-transfected Isl1^+^Sca1^+^ckit^+^ pCPCs also had a higher BNP expression (862.2 ± 450.5 pixels/cell; *p* < 0.05; Figure 6B,E) but was significantly lower than that of the Ang II hypertrophic cells.

### 3.6. ET-1 and Ang II Induced Hypertrophy Leads to Overexpression of MEF2c and GATA4 in Isl1^+^Sca1^+^ckit^+^ pCPCs

Gene expression analysis was executed with a real-time quantitative PCR cycler. The relative gene expression was calculated with a mathematical model that Pfaffl et al. previously described [56]:

(1) $$ratio=\;\frac{\;{\left(E_{target}\right)}^{\Delta CP\;target\;\left(control-sample\right)}\;}{{\left(E_{ref}\right)}^{\Delta CP\;ref\;\left(control\;–\;sample\right)}}.$$

**Figure 7:**
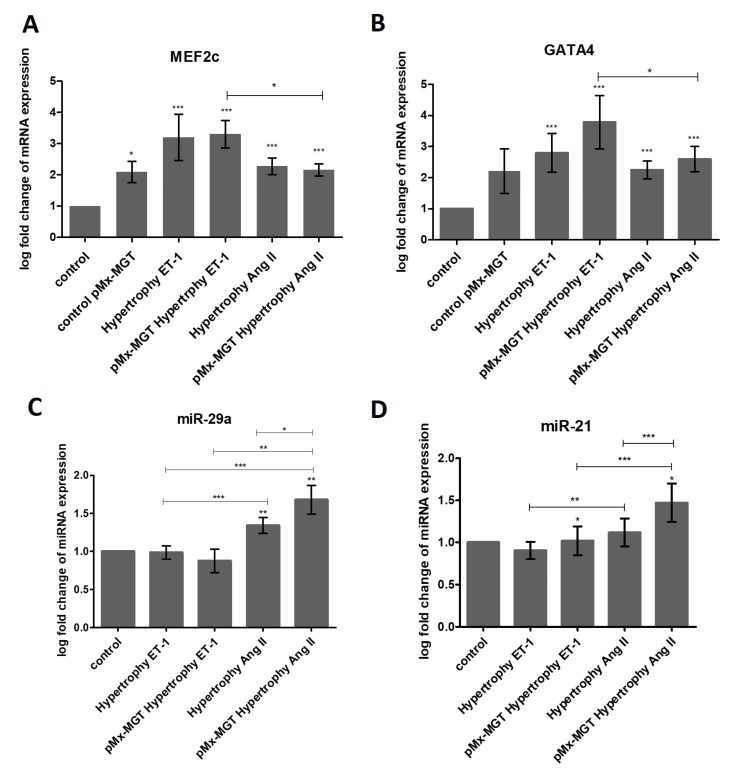
Gene expressions of MEF2c (**A**) and GATA4 (**B**) were significantly upregulated in all treatment groups (*p* < 0.005) with differences between Ang II and ET-1 (*p* < 0.05). MEF2c and GATA4 both were higher in the ET-1 groups. (**C**) miR-29a was significantly upregulated in both Ang II treated groups (*p* < 0.05), and (**D**) miR-21 was upregulated when cells were previously transfected with *pMx-MGT* in the Ang II treatment group. *pMx-MGT* hypertrophy ET-1-treated cells were downregulated in miR-21 expression compared to control group (*p* < 0.02).

The log-fold change of the target gene (e.g., MEF2c) was calculated by dividing the PCR efficiency of the target gene (MEF2c) raised to the power of the target gene ∆CP values (CT value of control group minus CT value of treatment group/sample) by the PCR efficiency of the reference gene (ACTB) raised to the power of the reference gene ∆CP values (CT value of control group minus CT value of treatment group/sample). The PCR efficiency was calculated with a standard curve and should have an ideal value of 2. As depicted in Figure 7A, the expression of MEF2c was significantly upregulated in all treated groups (all *p* < 0.0002). *pMx-MGT*-transfected pCPCs showed a log-fold change of 2.093 ± 0.8325 compared to control cells, which was 1. The gene expression log-fold change in ET-1-stimulated hypertrophic pCPCs was 3.203 ± 3.318, and it was 3.304 ± 1.971 in *pMx-MGT*-transfected ET-1-stimulated hypertrophic pCPCs. The log-fold change in Ang II stimulated cells was 2.279 ± 1.192, and in previously transfected *pMx-MGT* Ang II stimulated cells it was 2.160 ± 0.8665. There was also a significant difference between the *pMx-MGT*-transfected ET-1 treated cells compared to *pMx-MGT*-transfected Ang II treated cells (*p* < 0.05). This leads to the conclusion that ET-1 has stronger effects on MEF2c expression 24 h after hypertrophy stimulation than Ang II.

As expected, GATA4 expression was also significantly increased in all treated groups (*p* < 0.0004) compared to the control cells, with a log-fold change of 2.212 ± 1.756 in *pMx-MGT*-transfected pCPCs, 2.800 ± 2.776 in ET-1 treated pCPCs, 3.780 ± 3.836 in *pMx-MGT*-transfected ET-1 treated pCPCs, 2.244 ± 1.294 in Ang II treated pCPCs, and 2.595 ± 1.824 in *pMx-MGT*-transfected Ang II treated pCPCs. Again, ET-1 had a bigger effect on the expression of the GATA4 gene than Ang II (*p* < 0.003).

### 3.7. miR-29a is Upregulated in Response to Ang II Stimulation

We discovered a higher gene expression of miR-29a in Ang II treated cells but not in ET-1 treated cells (Figure 7C) compared to the control (*p* < 0.005). The log-fold expression change was 1.342 ± 0.4723 in Ang II treated pCPCs and 1.680 ± 0.8467 in *pMx-MGT*-transfected Ang II stimulated pCPCs, revealing a higher miR-29a expression in cells that were previously transfected (*p* < 0.05). There were also significant differences between the treatment groups displayed in Figure 7C.

### 3.8. pMx-MGT-Transfected Ang II Stimulated Isl1^+^Sca1^+^ckit^+^ pCPCs Have a Significanly Higher miR-21 Gene Expression

Furthermore, miR-21 expression was evaluated compared to the normal pCPCs (Figure 7D). There was an increase in Ang II hypertrophic treated cells when they were previously transfected with the cardiac reprogramming gene *pMx-MGT*. miR-21 expression in *pMX-MGT* hypertrophy ET-1 cells was significantly downregulated compared to control cells (*p* < 0.02). *pMX-MGT* ET-1 hypertrophic cells exhibited a log-fold change of 1.018 ± 0.7709, and *pMx-MGT* Ang II hypertrophic cells had a log fold change of 1.471 ± 1.027. There was a significant difference in miR-21 expression between those two groups (*p* = 0.0001).

## 4. Discussion

In contrast to the earlier assumption that mature cardiomyocytes cannot renew themselves, a lot of research has been done in recent years on CPCs, which have been shown to differentiate in vitro into cardiomyocytes and other cells of the cardiac cell line [8,9,57,58,59]. The role of CPC in the course of human life in cardiac disease is, therefore, not to be ignored, and it is important to evaluate changes on the cellular level resulting from disease more closely. Our goal was to establish a pCPC culture for the further evaluation of their relevance in cardiac hypertrophy. This would also enable testing future drugs before they are used in in vivo animal models.

We successfully isolated CPCs from porcine hearts that can be easily expanded in culture. They exhibit stem cell like characteristics with a spindle shaped morphology and cardiac progenitor cell markers such as Isl-1, Sca-1, c-kit, and αSMA as well as cardiac lineage associated markers such as Cx43, cTNT, and BNP. It has been previously reported that human CPCs express several markers such as c-kit, Isl-1, Sca-1, MDR1, CD31, and ki67 and cardiac lineage markers such as GATA4, MEF2c, Cx43, αSA, cTnI, and Nkx2.5 [60,61,62,63,64]. Furthermore, expression of BNP was observed in human c-kit positive CPCs in consequence to the induction of cardiac reprogramming with cardiac transcription factors [65].

In this study we stimulated pCPCs with angiotensin II or endothelin-1, both activators of hypertrophic growth by binding GATA4 in cardiomyocytes, to mimic hypertrophic pathways [15,43,44,46]. ET-1-treated human iPSC-derived endothelial cells have been shown to exhibit an impaired tube formation, impaired autophagy, and increased mitochondrial fragmentation in a high-glucose setting, which can be linked to ischemia-induced cardiac disease [66].

We could successfully demonstrate that *GATA4* gene expression was upregulated in hypertrophic pCPCs. We transfected pCPCs with a cardiac reprogramming plasmid (*pMx-MGT*) to stimulate development into cardiac myocytes. GATA4 expression was slightly enhanced in these cells, which could be explained by the assumption that GATA4 expression is naturally higher in cardiomyocytes than in cardiac progenitor cells. Likewise, MEF2c is known to be involved in the development of cardiac hypertrophy [27], which could be verified by our gene expression results. There was a significant upregulation in all hypertrophy-stimulated cells with a higher expression in ET-1-treated cells. ET-1 was described to activate MEF2c expression in the neural crest of mouse embryos [67]. Since Isl-1 pCPCs originate from the neural crest [9], a connection could be drawn. In addition, it has been reported that the transcription factor MEF2c can be specifically targeted by miR-21 in human neurons [68], which led us to investigate the gene expression of miR-21. Furthermore, we also measured miR-29a gene expression. The results of our experiments showed that miR-21 was significantly elevated only in transfected Ang II hypertrophic pCPCs. Wang et al. reported that an angiotensin II receptor blocker could inhibit miR-21-induced cardiac fibrosis [69]. Another team of researchers discovered that miR-21 gene expression was higher after stimulation with Ang II in rat neonatal cardiac fibroblasts [70]. But also, other cell types such as hepatic stellate cells exhibit higher miR-21 expressions when treated with angiotensin II, which seems to be involved in hepatic fibrogenesis pathways [71]. This leads to the conclusion that miR-21 is involved in the development of fibrosis through angiotensin-associated pathways, but it is not restricted to cardiac lineages. Our investigations show that miR-29a upregulation in pCPCs seems to be associated with angiotensin II stimulation but not with endothelin-1 stimulation. Our results indicate that the transfected and un-transfected pCPCs exhibit a significantly higher upregulation upon angiotensin II stimulation, compared to control and ET-1-stimulated pCPCs. Other documented results by Shi et al. showed similarities in the upregulation of miR-29a in Ang II hypertrophic rat cardiomyocytes, which they explained by its involvement in the PTEN/AKT/mTOR signaling pathway and inhibition of autophagy [38].

Besides gene expression changes, we could also observe changes in cell size. Hypertrophy was successfully induced, documented with an increased mean cell area in all treated cells. Interestingly, *pMx-MGT*-transfected cells had a higher increase in cell area than hypertrophy-stimulated pCPCs. This can be explained by the fact that cells increase in size during cardiomyocyte development [72,73,74], and stimulation with hypertrophic agents could lead to a delay in hypertrophic growth. Nevertheless, there was a significant change to control cells, which proves the efficacy of our hypertrophy model.

MCP-1 appears to play an important role in the course and development of many cardiac diseases, such as arteriosclerosis [75,76,77]. MCP-1 signaling can be initiated by Ang II, through NADPH activation and reactive oxygen species production, which further activates nuclear factor-kB (NF-kB) [78,79,80,81]. Intracellular MCP-1 protein changes in response to hypertrophy were substantiated through immunofluorescence mean integrated intensity quantification. We observed that MCP-1 was upregulated in transfected pCPCs as well as in Ang II stimulated pCPCs. However, the combination of *pMx-MGT* transfection and hypertrophy simulation seems to inhibit MCP-1 expression. This leads to the assumption that MCP-1 is decreased in early hypertrophic cardiomyocytes but upregulated in hypertrophic pCPC. The gap junction protein Cx43 is found all over the heart and plays an essential role in cardiac development and function [82,83]. Reduced expression of Cx43 is often associated with progressed heart disease such as chronic heart failure. In contrast, it has been shown that, in the initial phase of cardiac hypertrophy, Cx43 is upregulated [84,85,86]. We observed that Cx43 was upregulated in *pMx-MGT*-transfected cells and in combination with Ang II stimulation. These results correlate with previously documented findings that Ang II increases intracellular Cx43 expression in rat cardiac fibroblasts [87] and rat ventricular myocytes [88]. We hypothesize that transfection with cardiac reprogramming genes led to a successful initiation of cardiac differentiation with increased Cx43 levels, which was further maintained during Ang II stimulation but was blocked by ET-1. Spinella et al. described a similar effect using ET-1 in human ovarian carcinoma cells [89]. In our experiment, Cx43 was not upregulated in Ang II stimulated pCPCs, which indicates that pCPCs are somehow resistant to Ang II induced changes in gap junction protein expression. BNP is expressed in cardiomyocytes and is well known to be upregulated in cardiac hypertrophy; therefore, it is commonly used as cardiac biomarker [54]. BNP expression in our cells further validated our hypertrophy model because transfected pCPCs had a higher BNP expression than control pCPCs and an even higher expression after hypertrophy

## 5. Conclusions

In summary, pCPCs are a suitable cell type to study the effects of hypertrophy on both differentiated and undifferentiated pCPCs in vitro. Differentiated pCPCs react with typical signaling pathways that have already been demonstrated in human, rat, or mouse cardiomyocytes, such as changes in BNP, Cx43, and MCP-1 protein expressions or GATA4, MEF2c, and miR-29a gene expressions, but gene expression of miR-21 and protein expression of Cx43 does not appear to be upregulated in undifferentiated pCPCs in response to hypertrophic stimuli. This model offers great opportunities to further study basic disease mechanisms and potential progenitor cell effects in regenerating the diseased heart. Future medications could be tested in vitro before they are evaluated in the porcine translational model.

## Figures and Tables

**Table 1:** Primer list of genes used for pCPC characterization. Housekeeping genes are abbreviated with HK.

Gene	Forward Primer	Reverse Primer
ISL-1	ACATGACGGTGGCTTACAGG	ATGTCACTCTGCAAGGCGAA
SCA-1/Ly6a	AGCTCAGGGACTGGAGTGTT	ATCAGGGTAGGGGCAGGTAA
KIT (c-kit)	GCCTGTGACTATCTGGGCTC	GCCTTATTCATGCCCGGAGA
BNP	CGCAGTAGCATCTTCCAAGTC	ACCTCCTGAGCACATTGCAG
ACTB (HK)	TCAACACCCCAGCCATGTAC	CTCCGGAGTCCATCACGATG

**Table 2:** Primer list of genes used for mRNA and miRNA gene expression to evaluate hypertrophy stimulation. Housekeeping genes are abbreviated with HK.

Gene	Forward Primer	Reverse Primer
MEF2c	TAACATGCCGCCATCCGCCC	ATCCTCTCGGTCGCTGCCGT
GATA4	AGAAAACGGAAGCCCAAGAAC	CCACACTGCTGGAGTTGCTG
miR-29a	CGGACCTAGCACCATCTGAA	miScript Universal Primer (Qiagen)
miR-21	CGTAGCTAGCTTATCAGACTG	miScript Universal Primer (Qiagen)
ACTB (HK)	TCAACACCCCAGCCATGTAC	CTCCGGAGTCCATCACGATG
let7a (HK)	GCAGTGAGGTAGTAGGTTGT	miScript Universal Primer (Qiagen)
